# The UK’s 100,000 Genomes Project: manifesting policymakers’ expectations

**DOI:** 10.1080/14636778.2017.1370671

**Published:** 2017-09-06

**Authors:** Gabrielle Natalie Samuel, Bobbie Farsides

**Affiliations:** ^a^ Brighton and Sussex Medical School, University of Sussex, Falmer, UK

**Keywords:** Genomics, genome sequencing, genetics, expectations, genohype

## Abstract

The UK’s 100,000 Genomes Project has the aim of sequencing 100,000 genomes from UK National Health Service (NHS) patients while concomitantly transforming clinical care such that whole genome sequencing becomes routine clinical practice in the UK. Policymakers claim that the project will revolutionize NHS care. We wished to explore the 100,000 Genomes Project, and in particular, the extent to which policymaker claims have helped or hindered the work of those associated with Genomics England – the company established by the Department of Health to deliver the project. We interviewed 20 individuals linked to, or working for Genomics England. Interviewees had double-edged views about the context within which they were working. On the one hand, policymakers’ expectations attached to the venture were considered vacuous “genohype”; on the other hand, they were considered the impetus needed for those trying to advance genomic research into clinical practice. Findings should be considered for future genomes projects.

## Introduction

The United Kingdom’s (UK) 100,000 Genomes Project aims to sequence 100,000 genomes from UK National Health Service (NHS) patients who have a rare disease, an infectious disease, or cancer by 2017. Launched in 2012, its central goal is to implement genomics innovation/testing on a national scale such that it becomes routine in NHS practice. Alongside this, it also has a research goal to provide data for scientific discovery, making it the first ever research–clinical hybrid project within an NHS.^1^ The project has been described by policymakers as “revolutionis[ing] [the] fight against cancer and help[ing] 100,000 NHS patients” (Gov.uk. 2012) with “the potential to transform the future of healthcare … ” (NHS England 2014; our underline).

Such hyped descriptions of genetic/genomic innovations are not new. Scholars report a general “genetic optimism frame” within media and political representations of such technologies, which is now commonly referred to as “genohpye” (Nelkin 1995; Petersen 2001; Nerlich, Dingwall, and Clarke 2002). Such hype has been shown to over-emphasize the benefits of genetics/genomics through “breakthrough narratives,” and underrepresent the risks (Nerlich, Dingwall, and Clarke 2002; Bubela and Caulfield 2004; Henderson and Kitzinger 2007). Some social scientists have argued that such reporting has led to broken promises and hopes for the public and patients, and a public mistrust of genetic innovation and science, more generally (Fitzgerald 1998; Zurr and Catts 2005). Other social scientists – those exploring the sociology of expectations and those exploring related notions of promissory discourses – have shown how this hype is not simply a by-product of innovation, but rather constitutes the innovation process itself (van Lente 1993; Michael 2000; Fortun 2001, 2005, 2008; Brown and Michael 2003; Borup *et al*. 2006; Hilgartner 2015). By envisaging futures in the present using breakthrough narratives and future expectations, it creates a shared positive vision of the technology. This acts performatively by prompting alliance building and securing funding. In essence, hype associated with biomedical technology acts as a currency of “promissory capital” to drive the genetic bioeconomy and generate funding opportunities (Fortun 2001, 2005, 2008; Thompson 2005; Petersen and Krisjansen 2015; Joyner, Paneth, and Ioannidis 2016). The performative effects of previous genohype could, in fact, be argued to have played a role in the launch of the UK’s 100,000 Genomes Project.

The 100,000 Genomes Project has promised to transform the NHS by 2017. It has sufficient funding, and the Department of Health has specifically established an organization – Genomics England – to ensure its goals are achieved. We wanted to explore Genomics England, and in particular how policymakers’ expectations associated with the project have helped or hindered those trying to deliver the project into clinical practice – especially since clinical implementation projects are not always easy to implement “on the ground” (Rogers 2003; May and Finch 2009; Peters *et al*. 2013; Williams 2014; Day *et al*. 2016; Manolio, Ward, and Ginsburg 2016; The National Academies of Sciences 2016). We were not necessarily interested in the clinical implementation process itself, or providing a deep description of what was happening in clinical practice, but rather how the company set up to deliver the project, Genomics England, was functioning under the umbrella of policymakers’ expectations.

Using an interview methodology, in 2016, we explored the views and experiences of 20 individuals associated with, or working for Genomics England. We discovered interesting views within the group regarding the extent to which they felt helped or hindered by the “genohyped” context within which they were operating and we discuss these. We believe these findings will be useful considerations for any future genomes projects within the UK or internationally. Before doing so we provide a detailed overview of the 100,000 Genomes Project. To date, the venture has been described in little detail in the literature.

### The 100,000 Genomes Project

#### The venture’s launch

Decades of promissory discourses surrounding genetics and genomics have situated this field at the heart of many political health/research discussions in the UK, as well as internationally. Added to this, ex-UK Prime Minister David Cameron’s personal journey of having lost a son due to a genetic condition meant that the political climate in the UK was perhaps more open and willing to fund such promissory discourses into action. Thus, in 2009, and as part of the long political drive to implement genomics into healthcare, the UK House of Lords Science and Technology Committee called for the development of a “strategic vision for genomic medicine in the UK.”^2^ In response, the Government established the Human Genomics Strategy Group designed to monitor advances in genomics and develop a vision for the discipline within the NHS. The Strategy’s 2012 report explained the various steps needed to streamline genomics within the NHS and as such laid the foundations of the 100,000 Genomes Project (Human Genomics Strategy Group 2012, 9). Soon after the publication of this report, the venture was launched with the advertised aim to sequence 100,000 genomes of NHS patients, focusing on rare disease, cancer, and infectious disease. Its central goal is to implement genomics innovation on a national scale such that it becomes routine in NHS practice, though it also has an additional research goal (i.e. it is an innovative clinical–research hybrid project). The project received over £200 million in initial investment (Monitor Deloitte 2015).

#### More than just whole genome sequencing

While the Genomes Project bore out of a desire to bring patient benefit, to improve NHS infrastructure, and to drive research in the genetics arena, it also has the aim of kick-starting the development of a UK genomics industry.^3^ Thus, as with many biotechnologies, the project was “justified in terms of [its] potential to generate economic value” and exemplifies the “bioeconomy” at play (Petersen and Krisjansen 2015, 30). Indeed, the venture can be directly viewed as the realization of the Government’s 2003 vision to lead the world in harnessing the potential of genetics in healthcare (Department of Health 2003), and as such is expected to create opportunities for the genomics industry (Monitor Deloitte 2015). These opportunities are anticipated to come from services related to the Genomes Project such as the sequencing of the DNA, the data analysis, and the clinical interpretation. It is also foreseen that as the scale of genomes data grows, analysis and interpretation services will be in high demand, driving investment in areas such as analytics, data management, and data storage. As the Office for Life Science’s *Genomics in Industry* report has noted, the UK currently contributes 10% to the £8 billion global genomics market and it is anticipated that the genomes venture will double this proportion. The project is therefore much more than a DNA sequencing project. Moreover, it is about implementing the sequencing and interpretation of genomics into the NHS not only to bring patient benefit, but also for this benefit to be realized in terms of economic and industrial “bio-value” (Rose 2001; Waldby 2002; Petersen and Krisjansen 2015).

#### The National Health Service

Genomics England is the limited company wholly owned by the Department of Health tasked with carrying out the rare disease and cancer arm of the Genomes Project, though it is the UK’s NHS which has been tasked with delivering the venture. As such, the NHS has been deemed responsible for recruiting and consenting patients to the project, capturing clinical information about patients to inform DNA sequence analysis, and collecting and handling patients’ DNA samples prior to analysis. The latter function includes the processing of DNA samples: DNA extraction; barcoding, handling, identification; transport and transfer of samples; and sample storage. In order to fulfill its mission, in 2014 NHS England announced the formation of 13 Genomics Medicine Centres (GMCs) throughout the UK. These centers, each constituted of a number of alliance hospitals and organizations, were selected because of their track-record of providing excellence in genomic services. Their aim is to bring transformational change to the infrastructure of genomic medicine care delivery by delivering an end-to-end genomic medicine pathway. In spite of this, at the time of writing this paper (beginning of 2017), Genomics England/NHS were behind in their recruitment, with just over 17,000 genomes having been sequenced and far, far fewer sequences being analyzed or results returned. Our findings in this paper provided some insight into the reasons behind this.

#### Commercial and academic partners

While the NHS is tasked with the delivery of the genomic vision, Genomics England is responsible for procuring the sequencing capacity, the data architecture, and the necessary tools to securely store and interpret the 100,000 sequences and allow access for clinicians and researchers.^4^ To do this, Genomics England has formed a number of partnerships with a range of commercial and academic partners. For example, to sequence the DNA, Genomics England formed a partnership with the Wellcome Trust, The Wellcome Trust Sanger Institute and Illumina to create a Genomic Medicine Sequencing Centre in Hinxton,^5^ near Cambridge, UK. In terms of data analysis and interpretation, Genomics England has established the Genomics England Clinical Interpretation Partnership (GeCIP) of more than 2000 funders, researchers, trainees and clinicians from across the UK and the world. One of the main purposes of this partnership is, via carefully controlled access to the data, to allow those within the partnership to conduct beneficial and useful clinical research on the genomic data. This partnership also has the broader role in health professional education and training. As yet, the partnership is not up and running to its full potential, and it is still too early to tell whether this framework for analysis will be successful in the future. Genomics England has also established the Genomics Expert Network for Enterprises (GENE) Consortium – a selection of industry partners who are allowed access to Genomics England’s data during an industry trial throughout 2015. In this consortium, Genomics England remains the owner of the genomic sequences (along with the consequent legal and ethical consequences of this) and members are expected to pool knowledge and share the results of their analysis.^6^ However, again, little is known about the outcome of this trial or whether this approach to interaction with industry has been productive. Moreover, little is yet known about how the ethical issues associated with partnering with commercial entities might unfold. Though Genomics England does have an independent Ethics Advisory Committee to aid with ethical decision-making in relation to Genomics England’s policies and practice.

## Methods

### Rationale

As a medical ethicist and a medical sociologist working in the field of ethics, we were particularly interested in exploring how ethical issues associated with the Genomes Project have been, and are being, addressed by individuals on its Ethics Advisory Committee, as well as by those involved in the policymaking aspect of the initiative more broadly. We conducted 20 interviews with members of the Ethics Advisory Committee, the board of Genomics England, staff members, and those more broadly associated with the project to explore these issues. Some interesting insights emerged from our data with relation to how “ethics” was framed and performed within the organization. However, increasingly, during the course of our interviews, it was striking how much of the participants’ narratives revolved around the policymakers’ expectations of the project. It is these findings which are reported here. This is in line with the similar experiences of Bobbie Farsides, in which during a project exploring “new genetics” in ante-natal care, health care professionals were more interested in talking about issues relating to Down’s Syndrome.

### Recruitment

Potential respondents were recruited in the summer of 2016, at the approximate half-way mark through the 100,000 Genomes Project. Invites requesting participation in the research study, including participant information sheets, were emailed to individuals associated with, or those who worked for, Genomics England. Individuals from the following categories were invited: Genomics England staff members; Genomics England board members; Ethics Advisory Committee members; representatives from the Department of Health, Public Health England, NHS England, GMCs; and those involved in the evaluation of the 100,000 Genomes Project. Board member Professor Mike Parker and Head of Ethics, Laura Riley, assisted with recruitment by providing the names and email addresses of relevant individuals. Individuals were requested to respond to the email invite if they were interested in participating in the research project, or if they had any questions. A maximum of two follow-up emails were sent to non-responding individuals.

### Interviews

Twenty individuals responded to the email invite and 20 semi-structured interviews were conducted. All of the categories of individuals listed in the above section were represented in the interviews (exact *n* numbers are not provided to protect confidentiality given the small-scale nature of the project). All participants signed a consent form prior to the interviews commencing. Interviews were conducted either by telephone or face-to-face (at a location chosen by the participant), lasted between 30 and 105 minutes, and were audio-recorded. The interview schedule was broad, asking participants about their background and their role associated with the 100,000 Genomes Project. Participants were also asked their views on the project; on its benefits (present and potential) and drawbacks; on any issues they had come across in relation to their role in the project and how these had been overcome; and on the project’s political basis, as well as its ethics and public engagement strategy. Interviews were transcribed either by Gabrielle Natalie Samuel or by an external transcribing service.

### Analysis

Analysis of interview data was approached using inductive reasoning employing the inductive approach of grounded theory (Strauss 1987; Charmaz 2006). The analysis (or coding) of data was based on two inter-linked rounds: overview analysis and detailed analysis (Strauss 1987). Overview analysis consisted of memo-making and broad coding. Extensive memo-making was employed by the interviewer directly after each interview. Broad coding proceeded by scanning the interview transcripts for relevant ideas and themes. Detailed analysis of the full transcripts occurred line-by-line using NVivo software. Coding was carried out via constant comparison, which was continual, rigorous and allowed for developing and refining of conceptual categories as theory was developed. Due to the limited number of individuals associated with Genomics England, and the need to protect confidentiality, comparisons between respondents from different institutions are not reported.

### Ethics approval

Ethics approval was granted by Brighton and Sussex Medical School Research Governance and Ethics Committee (RGEC). Reference 16/014/FAR.

## Findings

Interviewee’s narrated two accounts of policymakers’ expectations about the 100,000 Genomes Project. One account pointed to a wide-range of issues which were hindering the project’s implementation into clinical practice as an illustration of why policymakers’ aspirations to sequence 100,000 genomes by 2017 were overly optimistic. Their dialogues resonated with previous literature which we discussed earlier, that highlights the pitfalls of unfulfilled promissory “genohype” often associated with genetic/genomic innovation (Brown and Michael 2003; Borup *et al*. 2006; Petersen and Krisjansen 2015). In the other contrasting account, interviewees’ perceived the expectations attached to the 100,000 Genomes Project as mobilizing an impetus in their attempts to overcome a wide-range of issues which were hindering the project’s clinical implementation. Below we explore these two accounts in detail. We note that the accounts of each interviewee could be situated within either narrative, somewhere in between, or as a mixture of both. We are careful not to identify participants in terms of their organizations, but note that there was some, though little, in the way of distinction between where interviewee’s accounts sat within or between these two different narratives and their respective host Institutions or job roles.

### Over optimistic expectations and genohype

In our interviewees’ first account of the promises associated with the 100,000 Genomes Project, the expectations of policymakers were viewed as “optimistic” (interviewee 4) and “overpromised” (interviewee 20). Interviewees pointed toward the as yet under performance of Genomics England in achieving its goals (roughly 17% of genomes sequenced at time of interview, midway between the project). These feelings of over overpromise rested almost in their entirety on the short time scale with which the project needed to be completed. There was an awareness that while the relative ease with which genomes can be sequenced marked one of several spurs for the development of the project, sequencing 100,000 genomes was much more complicated than the mere act of DNA sequencing. Sitting on one side of this was a complexity to “making sense of it [the sequence]”: “you can churn out 3 billion base pairs for $500 probably, but how much does it cost to make sense of it” (interviewee 3). This was especially true given the dual research–clinical nature of this project and the fact that innovation was occurring alongside delivery – the technology to handle and interpret such a large genomic data set was being developed concomitant to the collection of samples. On the other side, and the side focused on within this paper, there was the necessity to recruit patients and collect samples. Here, interviewees explained that the expectations attached to the project were unrealistic because the delivery and implementation of the project was to be carried out by the NHS. As such, much of the success of the 100,000 Genomes Project was “dependent on hospitals” (interviewee 15), relying on them to recruit individuals and collect DNA samples. It was acknowledged that the promise of the genomes venture was entering an already socially organized environment (the NHS) with its own firmly embedded political interests, as well as organizational, social, professional and cultural norms (“there are lot of social and institutional and professional and cultural barriers in the way of it working” (interviewee 13)). Such norms involved differing expectations about genomics, the importance of the Genomes Project, and its associated aims. A realization existed that to achieve the transformational shifts within the time frames of the project, a change in the thinking of the NHS and its staff towards genomics, and specifically this project, was needed. Altering these norms and expectations to harbor the project was seen as one of the keys to the project’s success. This, however, was perceived as not easy, with interviewee 4 noting that “there is the problem about how you persuade clinicians to join in … [because] … it is very difficult to shift NHS clinicians to that [genomic England’s] position” (interviewee 4). Indeed, speaking about organizational shifts post sequencing, interviewee 10, for example, noted that unanswered questions remained about how such a “transformational” (interviewee 9) change of the NHS could occur:

Right from the start was … the knowledge that GEL [Genomics England Ltd] generated would inform clinical practice in the NHS, and that brings with it a number of organizational tensions … is the infrastructure of the NHS going to be capable of incorporating this knowledge? Will we have the ability to use it properly? … What’s the skill needs that we will need within the health service to be able to deliver genomic medicine quickly and equitably? So there are a huge number of issues that need to be resolved or need to be addressed for it to be successful

Such tensions were viewed as especially pertinent by many interviewees given the “cash-strapped busy NHS” (interviewee 11): “when you actually put yourself down in the trust with its own costs and constraints, and many of the trusts are under special financial measures, that’s not their key priority” (interviewee 12).

Interviewees’ accounts revolving around overpromise were therefore contextualized by the friction emerging between on the one hand those working at Genomics England needing to meet the grandiose expectations of the 100,000 Genomes Project set by policymakers, and on the other, the differing priorities, expectations, cultures, norms, and organizational landscape of the NHS as a ground for clinical implementation of the project: “I don’t think we knew when we started … how complex it would be to build the infrastructure … you could say we may have been naïve” (interviewee 4). Interviewees provided detailed examples to substantiate their views about policymakers’ unrealistic expectations and below we briefly note two such illustrative examples.

#### Examples of the types of issues faced during clinical implementation

The 100,000 Genomes Project requires that about 70,000 patients (100,000 sequences) be recruited to the project via the NHS. However, as all interviewees noted, this has not been an easy task for the cancer arm of the study (“cancer has been difficult..[..]..in cancer they are struggling” (interviewee 10)) due to the differing priorities, expectations and culture of the NHS. For example, for interviewee 11, the research–clinical hybrid nature of the project meant that, rather than falling on an employed research nurse, recruitment responsibilities fell onto frontline clinical healthcare professionals – a staff team which was already “stressed” and “busy,” and maybe not very interested in assisting the genomes venture achieve its goals and fulfill its expectations:

if you go along and say well this patient’s in a research study and that they need an extra two blood samples taken in half an hour, can you do that..[..].. if you’re in a stressed busy clinical environment; it’s easier to not ask the question …  (interviewee 11).

Compounding this was the fact that many on this staff team may have little interest in research, or pursuing research endeavors:

I don’t think it’s [research] is as embedded in the frontline NHS psyche as we like to think it is … .some nurses think that research is just and add on and a faff and they don’t want to worry about it. So I think there’s a culture change to be done … [..]..it’s back to anything that makes things a bit different for them. They’ve got their routine, just processes that they do (interviewee 11)

For interviewee 18, a different concern about the differing priorities of the NHS versus the Genomes Project was apparent. This interviewee viewed a potential gap between executive support for the venture, and those individuals who are actually responsible for NHS day-to-day frontline recruitment. And, as this interviewee explained, for the latter, many clinical healthcare professionals may have personal and financial interests elsewhere, within their own already secured separate research funding. As such, recruitment for the project might be de-prioritized:

clinicians that work for the local trust and whose own research grants come from [other research funding bodies] are simply saying “right, I’ve got this person to agree to a clinical trial; let’s not complicate the issue by throwing anything else in” (interviewee 18)*.*

This was also of institutional concern, where the already high prevalence of clinical trials occurring within clinical cancer centers meant that vested interests remained here as opposed to the new genomics project in terms of removing samples from patients:

A lot of the cancer trials currently going on require samples and there’s an issue about how big the samples need to be to satisfy both Genomics England’s requirement and Cancer Research UK’s requirements. So I think there’s an argument there about just how much flesh you can cut out of one human being; [and also] the clinical trial requires tissue samples to be taken in this way and to be preserved in that way, that does not fit Genomics England’s needs, fine, they’re not going to look into it (interviewee 18)

Beyond these issues with recruitment, for the Genomes Project to be successful once recruitment had occurred, DNA samples need to be collected from participants for sequencing. However, similar to previous issues noted in the literature (Lazaridis *et al*. 2014), technical issues have emerged with the collection of cancer samples for genomic sequencing because the way in which tumor samples are routinely stored for processing damages DNA. Interviewee 16 explained that changing the sample processing protocol meant a huge organizational, professional, as well as cultural shift within the NHS: “what we had to do is to reengineer the way the National Health Service does this across its 13 Genomic Medicine Centres … ” and as interviewee 7 noted, this meant “developing new approaches to the handling of tissue, and as soon as you’re saying you’re going to do that, your reforming processes deep at the heart of pathology across the NHS” (interviewee 7). Interviewee 17 advised that while such changes may not seem like “rocket science,” they are often the hardest to implement in practice because of differences in priorities and expectations within an already stretched NHS.

It’s not rocket science. It involves … steps like having someone on hand to carry the sample from the theatre to the path lab. Now, an actually busy hospital, just organizing that is not immediately obvious … it then can’t take two or three days to get to the path lab. It has to actually go there in a few hours … so all of that is in an NHS which is under pressure for all sorts of reasons, is not an easy thing to do … 

This change was perceived as particularly difficult and interviewee 4 was becoming increasingly frustrated that the NHS laboratories, who maybe did not share the vision of policymakers’ expectations about the Genomes Project, were opposing change to their “outdated model” of pathology testing:

I think that someone needs to take people that run genomics labs in the NHS by the scruff of their necks and shake them up to the fact that … . power comes from having high quality data that you use and that they are trying to keep to an outmoded model.

Overall then, the instances described above, and similar issues (including, but not limited the lack of resources and staff within the NHS to accommodate the transformation change, and the lack of education and support and to want to drive the change), were used by interviewees as illustrative exemplifiers of the overly optimistic expectations associated with the Genomes Project that it could fulfill its promises, and of the “underestimated” time (interviewee 9) provided with which to achieve said aims. Most of these instances can be tied together by the realization of a tension between the need for Genomics England to implement its aims, and the fact that the NHS as an organizational structure did not, and more importantly *could* not, always share the same vision of genomics. Indeed, the “cash-strapped” NHS had/has its own set of issues to face on a day-to-day basis far removed from the implementation of genomics. And interviewee 5 noted that whilst the project was not “misconceived, [it was] rather simplistically assumed how easy it would be.” Interviewee 12 stated that “a very bold statement [the 100,000 Genomes Project announcement], but in terms of delivery, it just makes it impossible to do.”

### Expectations and the mobilization of motivation

Interviewees also had an alternative account to the above perspective on policymakers’ expectations of the Genomes Project. From this second perspective, interviewees viewed the hype surrounding the venture as a mobiliser of the project. In this narrative, the project’s political foundations were seen to bring benefits: “the most difficult thing is that we are a political project, but that’s also the biggest advantage” (interviewee 2). This was because the time pressure, borne out of the need to meet the expectations attached to the project meant that “[the project] wouldn’t have happened probably as quickly without that policy support” (interviewee 11); “we are where we are and a lot quicker than we would have been” (interviewee 17). And while the fast-pace of the project meant that it was “all a bit of a learning process” (interviewee 9), with very little time to plan ahead (“we are building the airplane while we are flying it” (interviewee 15)) so that some mistakes were being made (“you make mistakes” (interviewee 17)), at the same time, it had its benefits. The speed was seen as “one of the ways to innovate really rapidly” (interviewee 16), and allowed years of “painful” planning to be avoided: “you ended up in these scenarios where people were trying to plan for every possible circumstance and it was incredibly painful and it was still chaotic at the end” (interviewee 2). As such, the political nature of the project, although causing friction, provided a “can-do attitude” (interviewee 1), and was “needed” to force paced decision-making: “I think the friction that has happened, the pace of things, I think that needed to happen because academics would always – they would take far too long to come to this decision” (interviewee 18). Interviewee 2, for example, spoke about the political rhetoric giving the project “impetus” to drive change:

the fact that we are a political project with a political view means that we have that focus to be concentrated on driving a lot of the change … there is a lot of political rhetoric around it because that’s what’s given it the impetus (interviewee 2).

In this account of policymakers’ promises, interviewees perceived the project’s tightly framed expectations and deadlines as working positively to help surmount the issues facing the project’s clinical implementation described above. This was because the speed with which the Genomes Project needed to work to meet such expectations was viewed as the necessary factor to drive change in NHS culture: “if you go to slowly you end up with compromise of the lowest common denominator. The objective was absolutely to drive this fast, to have the NHS and our researchers ahead … and not to give people time to downgrade it” (interviewee 4). As interviewee 17 remarked, the idea was that if the project – which needs to overhaul NHS services – moves fast enough, the NHS would not have time to “work out its defence” to any organizational, cultural, social, and professional changes required within the NHS, thus making any transformations easier:

If you’re not careful, you get caught up in normal human defence to change. If you move fast enough, you can, to a degree, minimize that [laugh] … I’m told by people who’ve been in the business much longer than I have that all previous attempts to reform pathology have failed. But we seem to be getting there. I think that’s possibly because we’ve arrived at such speed … we had to change before the system worked out its defence to that (interviewee 17)

Therefore, while the project did raise a range of clinical implementation issues, the alternative, that is, not implementing the project with the policymakers expectations attached, was not viewed as necessarily any better:

change is always painful [and] … I recognize the challenges [of having the political expectations tied into the project], but when people talk about alternatives, I think that they are perhaps being a little naïve in thinking that those alternatives would necessarily be better (interviewee 2)*.*

In fact, while the nature of the expectations meant that it was “uncomfortable” and “tough” for the workforce who were trying to implement the Genomes Project, it was the tight deadlined expectations themselves which, in essence, provided the motivation and momentum that was needed as they attempted to drive a cultural shift in priorities within the NHS:

I think it’s very uncomfortable for everybody … but I think it’s a very brave project and it is one way to get the NHS to change systems is just to say it’s changing and see how everybody innovates and how everybody works around that and I think that is the policy, that is the motivation. So I probably think it’s a good thing, a good thing for patients eventually … (interviewee 9)

This is not to say whether or not this expectation-driven motivation would lead to the project’s success, since we have no way of knowing this from this data, but rather there was a view that the policymakers’ expectations mobilized an “impetus” and motivation for individuals within Genomics England in their attempts to drive the project forward.

It is worth noting that having one entrepreneurial company – Genomics England, both responsible and answerable for meeting the expectations for the project’s eventual success, most likely facilitated the momentum/motivational affects of the policymakers’ expectations. Having this focal point meant that the impetus and drive could abound throughout the company, its staff and associated individuals. These could then be championed outside the project more broadly (Lazaridis *et al*. 2014; Kukk, Moors, and Hekkert 2016). Indeed, interviewee 3 felt that the status of Genomics England as an entrepreneurial company meant that in the face of tight deadlines and expectations, it was more likely than academics/scientists to adopt a can-do attitude in the face of trying to change NHS clinical culture:

it’s very difficult to get the [translational] stuff into the clinic, and I think part of the problem is science itself and scientists. They are not brave enough..[..]..the entrepreneur [Genomics England] … will take a judgment … and then will do something about it.

## Concluding remarks

This paper has explored how policymakers’ expectations manifested within the UK’s recent and ongoing 100,000 Genomes Project. Our interviewees’ narrated two accounts relating to this. The first emphasized the various understandable issues hindering the project’s implementation into clinical practice as an illustration of what interviewees viewed as the overly optimistic “genohype” associated with the project. In constructing this narrative, interviewees’ accounts reflected what is already known about the complex process of implementation (May and Finch 2009) and thus viewed the grandiose statements and promises affixed to the Genomes venture as “naïve” and “overpromised.” The second account perceived the expectations of the project, particularly given the time constraints attached to them, as acting as an impetus and motivator of Genomics England’s efforts to drive clinical implementation and delivery. This can be compared to previous work exploring the future-orientated expectations attached to other biomedical innovations/other genetic/genomic technologies, which have been shown to do performative work in the generation of funding, the promotion of alliance building and the allocation of resources (Hedgecoe and Martin 2003). Only here, in a company which has been already fully funded, the expectations are functioning as a stimulus for individuals within Genomics England, working to drive change.

The presence of these two different accounts of the 100,000 Genomes Projects’ political expectations immediately suggests a possible relevance of the work of Gilbert and Mulkay. These authors have drawn on extensive research in the field of biochemistry, and in particular oxidative phosphorylation, to argue that scientists’ descriptions of their actions and beliefs about science are context-specific. The authors note two predominant styles of interpretation of events: those in the formal context, based on an empiricist repertoire (i.e. often seen in academic journals – science as impartial; scientists are detached) and those in the informal context, based on a contingent repertoire (i.e. drawing on social, psychological and other factors), often used to explain away controversies or “others’” errors in science. Such discourses, say the authors, are reconciled by the belief that social distorting influences which cause errors and controversies in science will eventually be made apparent and science will prevail (Gilbert and Mulkay 1984). The authors argue that both discourses are equally important and valid, and rather than trying to combine them to form one interpretation of events (as is typically the case), they recommend different participants’ discourses be treated as a topic of analysis as a whole made up of different but not necessarily inconsistent parts.

Applied to our findings, this would suggest that interviewees’ discussions promoting the benefits of the political expectations attached to the Genomes Project reflect the formal, empiricist repertoire or version of events, whereas interviewees’ discussions about the difficulties faced by the project are contingent – interviewees maintain their own credibility and that of Genomics England by explaining away difficulties using “genohype” and other social/organizational issues such as those related to the need to rely on the NHS. This is an interesting way to consider our findings, and sits alongside the approach we have taken.

Moving beyond a discourse approach, some of our findings are not unexpected. Interviewees accounts of overpromise and issues with clinical implementation are unsurprising given that those who are involved in the project are likely to want to explain how hard the project has been, and express concern about being able to deliver the project – especially given the issue of resources within the NHS, a point further explored by Day and colleagues in their ethnographic study of a large, research intensive NHS breast cancer service implementing a stratified medicine approach (Day *et al*. 2016). More surprising were interviewees’ perspectives on the benefits of working under difficult time constraints – constraints which were also viewed as overpromised. One might expect that interviewees would only have negative things to say about working under these conditions (and a lot of them did indeed express the pressure which had been placed on the workforce). Yet this was not always the case, benefits were also noted. Talking to individuals at Genomics England suggested that when trying to make a big (national) shift from research to the clinic, working to tight deadlines, and working within a pressured environment, while “tough” and “uncomfortable”, was having a motivational role. This was seen as then acting to provide the momentum required by Genomics England in its attempts to drive change in clinical practice – a practice which is well known to be particularly resistant to such change and a phenomenon sometimes referred to as clinical inertia. This is not to say that the impetus and motivation generated from policymakers’ expectations will indeed bring about the project’s success – we have no way of knowing if the project will be successful from this data, and indeed (while not genome sequencing), Day and colleague’s ethnographic study suggests a whole range of issues affecting patient care during the implementation of a stratified medicine approach (Day *et al*. 2016). Rather, with well-acknowledged time constraints hanging over Genomics England, the expectations attached to the Genomes Project inadvertently acted as a currency of hype (Petersen and Krisjansen 2015) – a vision of what needed to be achieved. This then provided a stimulus, a perceived necessary factor, as Genomics England attempted to drive clinical change. This can be compared to other “currencies of hype” and promissory discourses disseminated by companies within the field of genomics, for example those associated with the synthetic biology “Biobricks group,” explored by Hilgartner (Hilgartner 2015), and those associated with deCODE Genetics – explored by Fortun (Fortun 2001). In the latter case, Fortun argued that the promises, hype, and “stories” promoted by deCODE genomics were integral to the science and business of the company, and that “to the extent that we can say it is ‘based’ on anything, the genomics industry is based on … what genetic information may become in the anticipated, contingent future … . there can be no economy without hype … ” (Fortun 2001, 145–146). While our findings suggest a positive role for the presence of such hype and promissory discourses, Fortun in comparison, comments that

six years of researching, speaking on, and writing about deCODE … here is a case so chock-full of false and broken promises … that I can confidently make the “forward-looking statement” that Kari Stefansson [CEO and founder of deCODE] has the least right to make promises of any genomics company CEO I know (Fortun 2005, 158).

Happily, the researchers in this instance were not compelled to reach a similar conclusion.

To summarize, then, these findings caution us not to view the genohype surrounding the venture as just vacuous hype, something which is hindering the project, or is problematic because it over promises what cannot be delivered. While the project may have been “overpromised” and “misconceived” (it is too early to tell), the promissory discourses attached to it have become a part and a drive of the project itself. Having an entrepreneurial Genomics England with full responsibility for achieving the project’s promised political goals as a focus for this drive and motivation, and having a specific time frame with which Genomics England needs to meet these expectations, has allowed the project to acquire momentum as Genomics England uses all its effort to enact change at the level of clinical delivery. Thus, while expectations and promissory discourses can lead to negative outcomes (as noted in relation to Fortun’s analysis of deCODE above (Fortun 2005)), it is important to recognize that this is not always the case. These findings should be considered during the development of any future genomes projects within the UK, and also internationally.

By way of limitations, we note that our interviewees were self-selecting individuals approached through a contact list provided by Genomics England. This self-selection of participants may have created a particular bias, and there may have been others at the company who would have provided valuable insight about the company who did not participate in the study. Furthermore, the interviews were conducted in 2016, before the 100,000 Genomes Project’s completion. As we have noted above, further research needs to be conducted to explore how the innovation process progresses; whether (and/or how) successful clinical delivery is implemented; and whether the promissory discourses associated with the initiative lead to real benefits, or whether these “communities of promise” – or “genohype” – fall apart and migrate to new areas, as described in the “sociology of expectations” (Michael 2000; Brown and Michael 2003; Borup *et al*. 2006).

## Disclosure statement

No potential conflict of interest was reported by the authors.

## Notes

1. Other research–clinical hybrid projects not on a national scale include, for example, the United States Geisinger health systems’ MyCode^®^ genomics data system and biobank, and the United States Mayo Clinic’s Center for Individualized Medicine. The research–clinical hybrid setup is interesting in terms of novel conceptions of innovation pathways and is the subject of a forthcoming paper (Dheensa *et al.*).

2.
http://www.publications.parliament.uk/pa/ld200809/ldselect/ldsctech/107/10711.htm.

3.
https://www.genomicsengland.co.uk/about-genomics-england/.

4.
https://quarterly.blog.gov.uk/2014/01/30/100000-genomes/.

5.
https://www.genomicsengland.co.uk/wp-content/uploads/2014/11/GeCIP-Guidance_FINAL.pdf.

6.
https://www.genomicsengland.co.uk/clinicians-researchers-and-industry-collaborate-with-the-100000-genomes-project/.
